# Supplementary figures and images for: A small set of conserved genes, including sp5 and Hox, are activated by Wnt signaling in the posterior of planarians and acoels
Source: PLoS Genet. 2019 Oct 18;15(10):e1008401. doi: 10.1371/journal.pgen.1008401 (PMC6821139; doi:10.1371/journal.pgen.1008401)

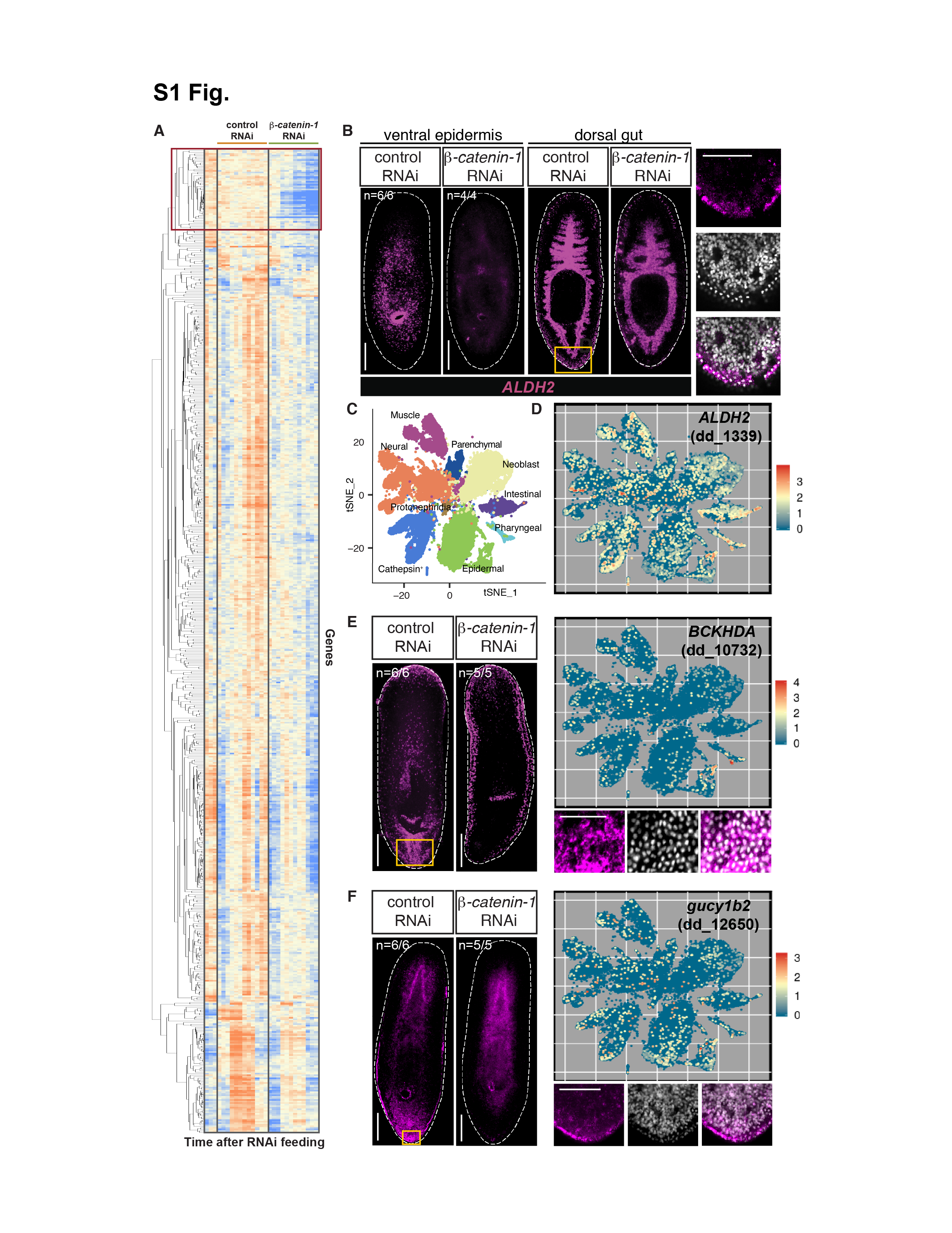

Supplement: S1 Fig — (A) Heatmap of genes down-regulated after β-catenin-1 RNAi at any timepoint (padj<0.05 and log2 fold change ≤-0.5) subjected to hierarchical clustering. Heatmap displays gene expression counts as z scores for time-points post-RNAi feeding. Red box indicates cluster shown in Fig 1B. Differential expression analysis provided in S1 Table. (B) Posterior epidermal expression of ALDH2 is β-catenin-1 dependent. FISH for ALDH2 (magenta) after control and β-catenin-1 RNAi. Left: Maximum intensity projection of ventral epidermis. Middle: Maximum intensity projection of dorsal gut. Scale bars 200μm. Right: Zoom in of tail tip indicated by yellow box showing ALDH2 expression in epidermis; ALDH2 (magenta), DAPI (gray). Scale bars 100μm. (C) Key for tSNE-plots displaying single cells. Major planarian cell types are labelled with distinct colors. (D) ALDH2 is expressed in many cell types. tSNE-plot of planarian cells colored by ALDH2 (dd_1339) gene expression (red, high; blue, low). (E) BCKHDA is expressed in posterior epidermis. Left: BCKHDA expression during homeostasis, 6 days post control and β-catenin-1 RNAi. Scale bars 200μm. Right top: tSNE-plot of planarian cells colored by BCKHDA (dd_10732) gene expression (red, high; blue, low). Right bottom: Zoom in of tail tip indicated by yellow box showing BCKHDA expression in dorsal epidermis; BCKHDA (magenta), DAPI (gray). Scale bars 100μm. (F) Posterior expression of gucy1b2 is β-catenin-1 dependent. Left: gucy1b2 expression during homeostasis, 6 days post control and β-catenin-1 RNAi. Scale bars 200μm. Right top: tSNE-plot of planarian cells colored by gucy1b2 (dd_12650) gene expression (red, high; blue, low). Right bottom: Zoom in of tail tip indicated by yellow box showing gucy1b2 expression in posterior epidermis and sub-epidermal cells; gucy1b2 (magenta), DAPI (gray). Scale bars 100μm. (TIF) [file pgen.1008401.s014.tif]

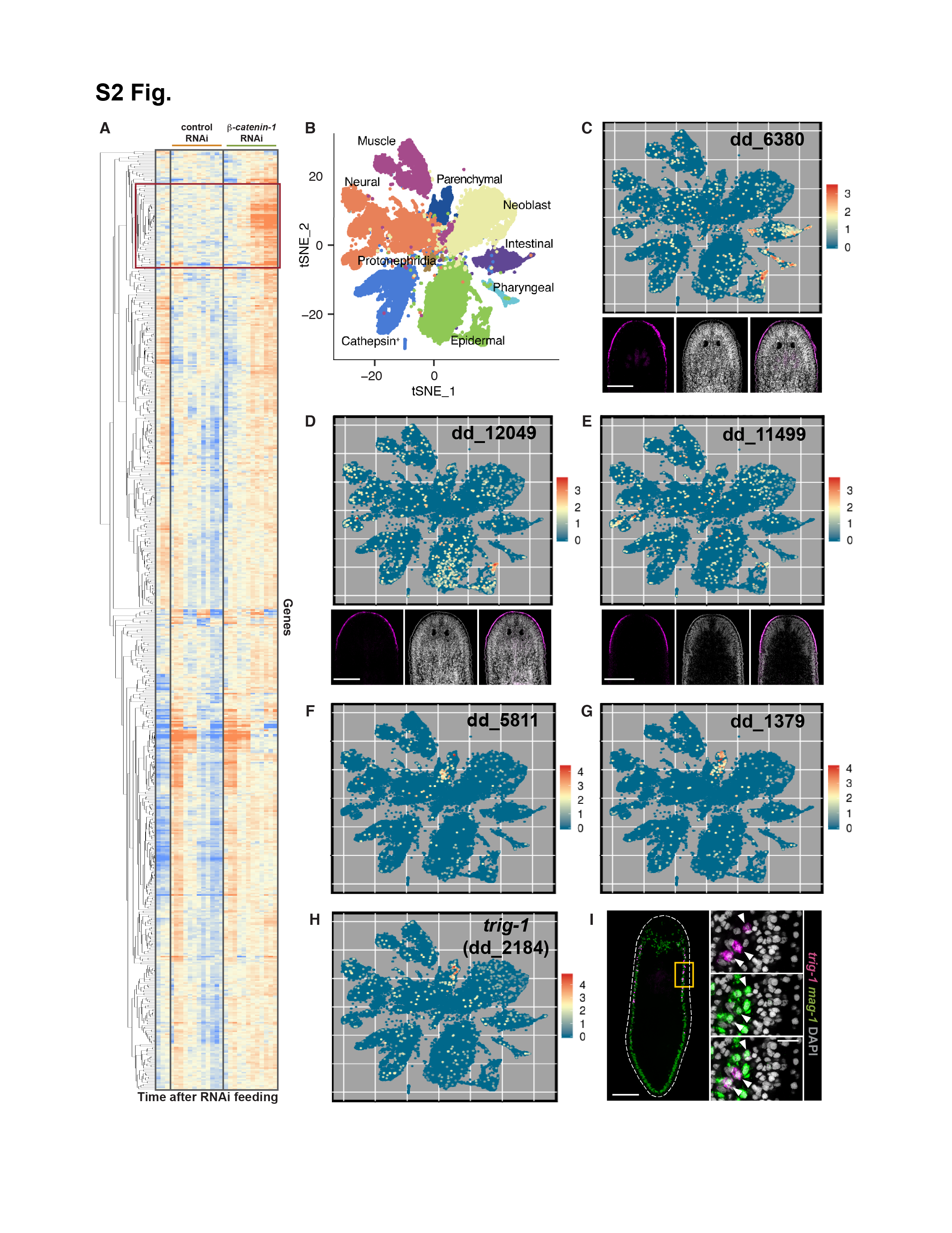

Supplement: S2 Fig — (A) Heatmap of genes up-regulated after β-catenin-1 RNAi at any timepoint (padj<0.05 and log2 fold change ≥0.5) subjected to hierarchical clustering. Heatmap displays gene expression counts as z scores for time-points post RNAi feeding. Red box indicates cluster shown in Fig 2B. Differential expression analysis provided in S1 Table. (B) Key for tSNE-plots displaying single cells. Major planarian cell types are labelled with distinct colors. (C) dd_6380 is expressed in anterior epidermis. Top: tSNE-plot of planarian cells colored by dd_6380 gene expression (red, high; blue, low). Bottom: Single dorsal confocal slice showing expression of dd_6380 (magenta) in the epidermis (DAPI, gray). Scale bar 200μm. (D) dd_12049 is expressed in anterior epidermis. Top: tSNE-plot of planarian cells colored by dd_12049 gene expression (red, high; blue, low). Bottom: Single dorsal confocal slice showing expression of dd_12049 (magenta) in the epidermis (DAPI, gray). Scale bar 200μm. (E) dd_11499 is expressed in anterior epidermis. Top: tSNE-plot of planarian cells colored by dd_11499 gene expression (red, high; blue, low). Bottom: Single dorsal confocal slice showing expression of dd_11499 (magenta) in the epidermis (DAPI, gray). Scale bar 200μm. (F) dd_5811 is expressed in parenchymal cells. tSNE-plot of planarian cells colored by dd_5811 gene expression (red, high; blue, low). (G) dd_1379 is expressed in parenchymal cells. tSNE-plot of planarian cells colored by dd_1379 gene expression (red, high; blue, low). (H) trig-1 is expressed in parenchymal cells. tSNE-plot of planarian cells colored by trig-1 gene expression (red, high; blue, low). (I) trig-1 is expressed in marginal adhesive gland cells. Left: FISH for trig-1 (magenta) and mag-1 (marginal adhesive gland cells, green) in an uninjured animal. Scale bar 200μm. Right: Zoom of pre-pharyngeal region marked by yellow box. Arrows mark co-expressing cells. Scale bar 20μm. (TIF) [file pgen.1008401.s015.tif]

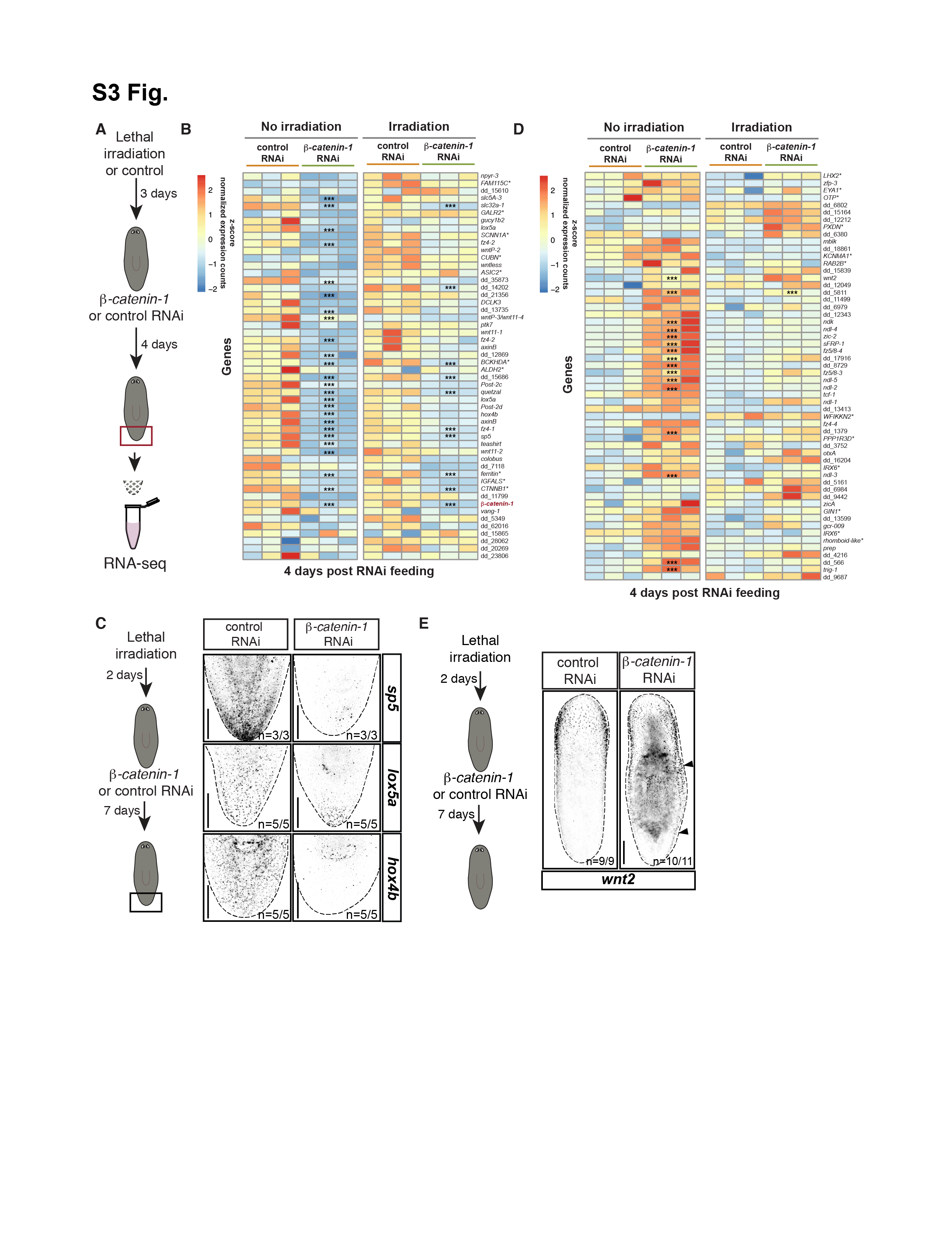

Supplement: S3 Fig — (A) Experimental scheme for RNA sequencing. Animals were subjected to lethal or no irradiation, followed by one RNAi feeding and collection of tails 4 days post feeding. (B) Heatmap of 52 genes identified in Fig 1B at day 4 post RNAi feeding for indicated irradiation conditions. Heatmap displays gene expression counts as z scores. *indicates annotation by best BLAST hit. ***padj<0.05. Differential expression analysis provided in S3 Table. (C) Down-regulation of planarian sp5 and Hox genes after β-catenin-1 RNAi is irradiation insensitive. FISH for sp5, lox5a, and hox4b after control and β-catenin-1 RNAi in irradiated animals during homeostasis. Images are presented in grayscale with color inverted. (D) Heatmap of 56 genes identified in Fig 2B at day 4 post RNAi feeding for indicated irradiation conditions. Heatmap displays gene expression counts as z scores. *indicates annotation by best BLAST hit. ***padj<0.05. Differential expression analysis provided in S3 Table. (E) Posterior expansion of anterior expression domains after β-catenin-1 RNAi is irradiation insensitive. FISH for wnt2 after control and β-catenin-1 RNAi in irradiated animals during homeostasis. Arrows indicate posterior boundary of gene expression. Images are presented in grayscale with color inverted. Scale bars, 200μm. (TIF) [file pgen.1008401.s016.tif]

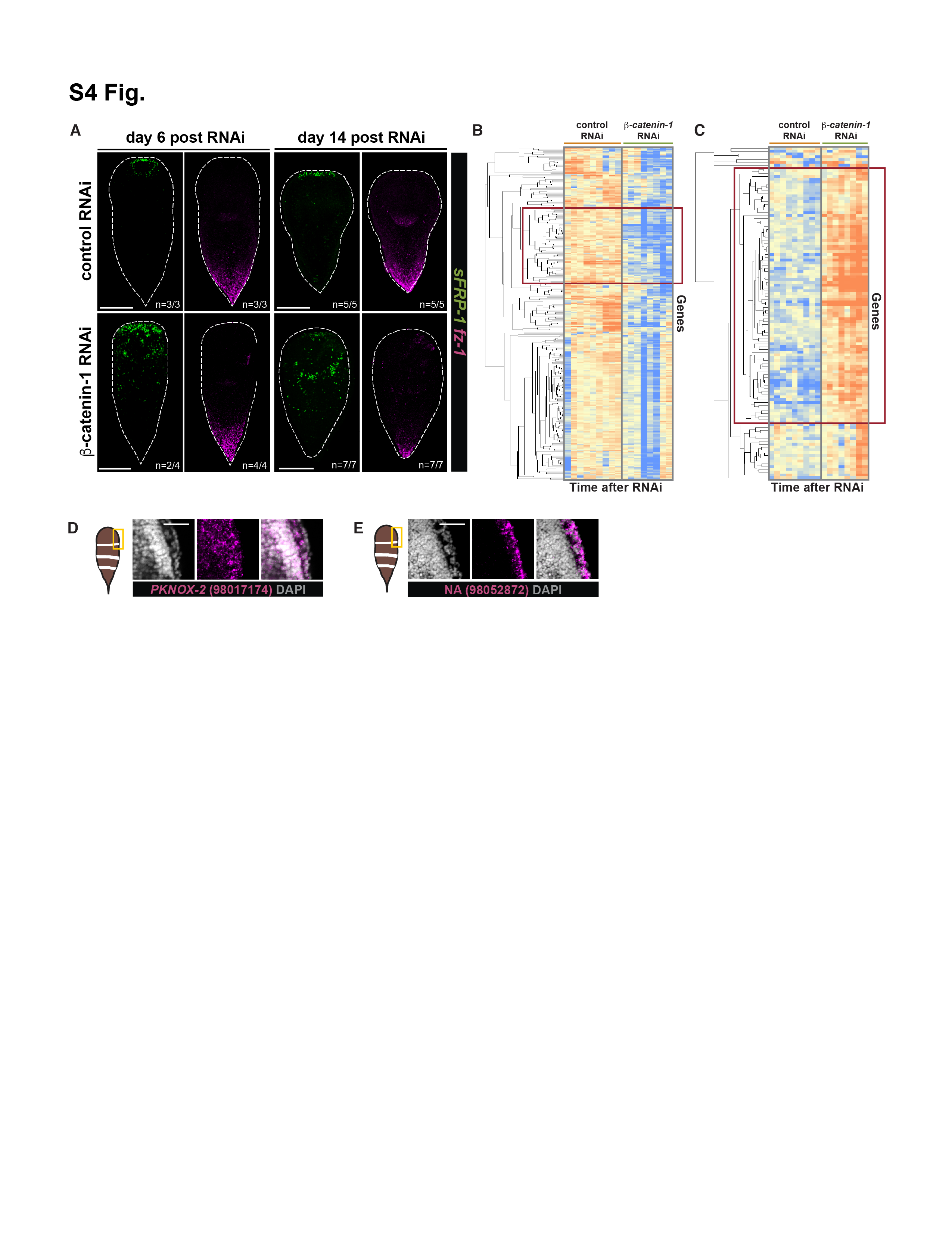

Supplement: S4 Fig — (A) Changes in PCG expression occur by day 6 post β-catenin-1 RNAi in Hofstenia miamia. FISH for anterior PCG sFRP-1 (green) and posterior PCG fz-1 (magenta) at indicated time points post RNAi initiation during homeostasis. Scale bars, 200μm. (B) Heatmap of all genes down-regulated after β-catenin-1 RNAi (padj<0.1) subjected to hierarchical clustering. Heatmap displays gene expression counts as z scores for time-points post-RNAi feeding. Red box indicates cluster shown in Fig 3B. Differential expression analysis provided in S4 Table. (C) Heatmap of all genes up-regulated after β-catenin-1 RNAi (padj<0.1) subjected to hierarchical clustering. Heatmap displays gene expression counts as z scores for timepoints post-RNAi feeding. Red box indicates cluster shown in Fig 4B. Differential expression analysis provided in S4 Table. (D) PKNOX-2 is expressed in anterior epidermis and sub-epidermal cells. FISH for PKNOX-2 (98052872) (magenta) with DAPI. Single confocal slice of anterior dorsal epidermis, region indicated by yellow box. Scale bar, 50μm. (E) 98052872 is expressed in anterior epidermis. FISH for 98052872 (magenta) with DAPI. Single confocal slice of anterior dorsal epidermis, region indicated by yellow box. Scale bar, 50μm. (TIF) [file pgen.1008401.s017.tif]

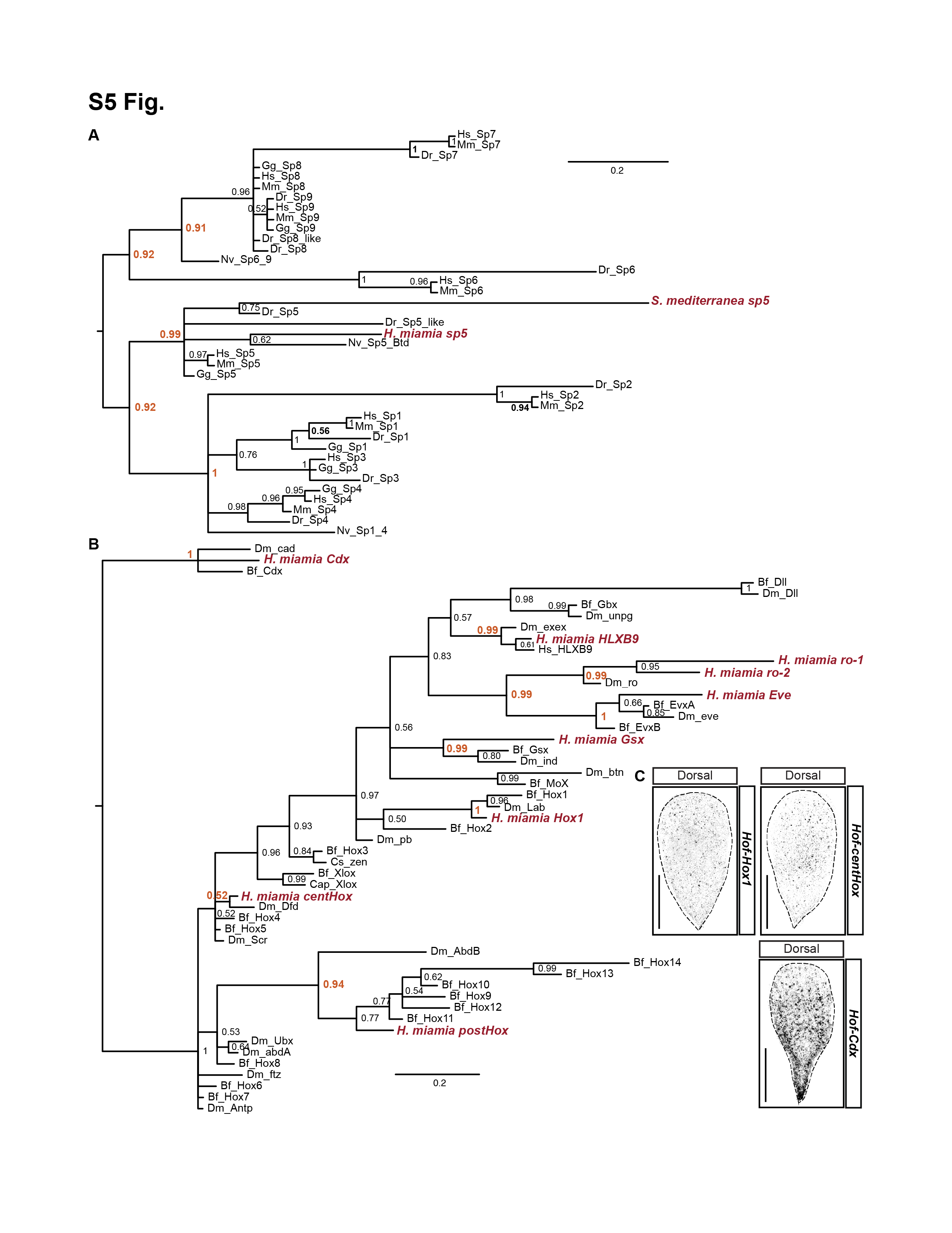

Supplement: S5 Fig — (A) Phylogenetic tree for the placement of Schmidtea mediterranea and Hofstenia miamia SP5. Bayesian analysis of SP family proteins. Posterior probability indicated at nodes. Hs (Homo sapiens); Mm (Mus musculus); Dr (Danio rerio); Gg (Gallus gallus); Nv (Nematostella vectenesis). Protein sequences provided in S6 Table. Nexus file provided as S1 Dataset. (B) Phylogenetic tree for the placement of Hofstenia miamia Hox proteins. Bayesian analysis of Hox and ParaHox proteins. Posterior probability indicated at nodes. Cap (Capitella teleta); Cs (Cupiennius salei); Bf (Branchiostoma floridae); (Homo sapiens); Dm (Drosophila melanogaster). Protein sequences provided in S6 Table. Nexus file provided as S2 Dataset. (C) FISH for Hof-Hox1, Hof-centHox and Hof-Cdx in two-week old hatchlings. Images are presented in grayscale with color inverted. (TIF) [file pgen.1008401.s018.tif]

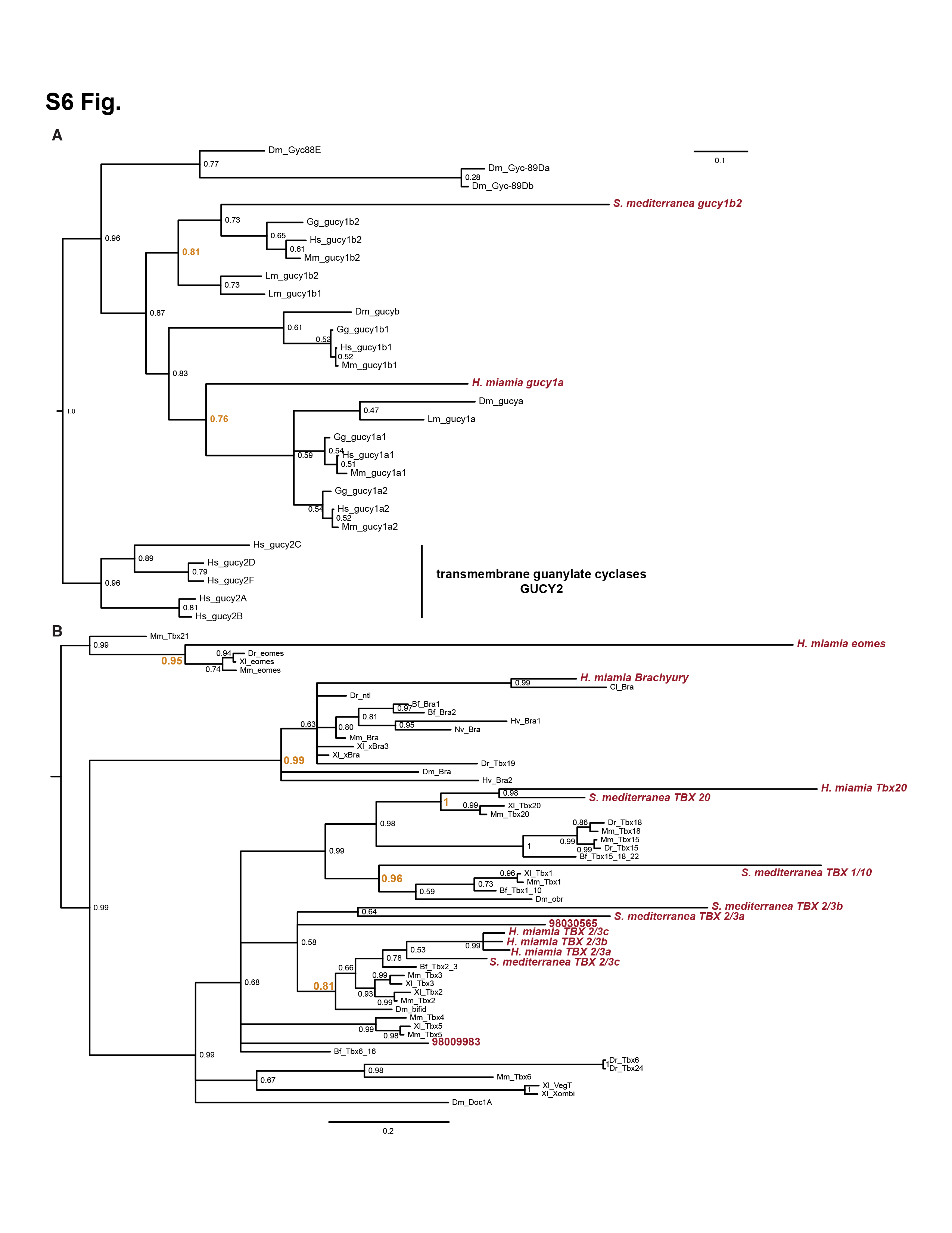

Supplement: S6 Fig — (A) Phylogenetic tree for the placement of Schmidtea mediterranea and Hofstenia miamia guanylate cyclase proteins. Bayesian analysis of soluble and transmembrane guanylate cyclase proteins. Posterior probability indicated at nodes. Hs (Homo sapiens); Mm (Mus musculus); Dm (Drosophila melanogaster); Gg (Gallus gallus); Lm (Limax marginatus). Protein sequences provided in S6 Table. Nexus file provided as S3 Dataset. (B) Phylogenetic tree for the placement of Schmidtea mediterranea and Hofstenia miamia Tbx proteins. Bayesian analysis of Tbx family proteins. Posterior probability indicated at nodes. Nv (Nematostella vectenesis); Xl (Xenopus laevis); Dr (Danio rerio); Hv (Hydra vulgaris); Dm (Drosophila melanogaster); Cl (Convolutriloba longifissura); Bf (Branchiostoma floridae); Mm (Mus musculus). Protein sequences provided in S6 Table. Nexus file provided as S4 Dataset. (TIF) [file pgen.1008401.s019.tif]

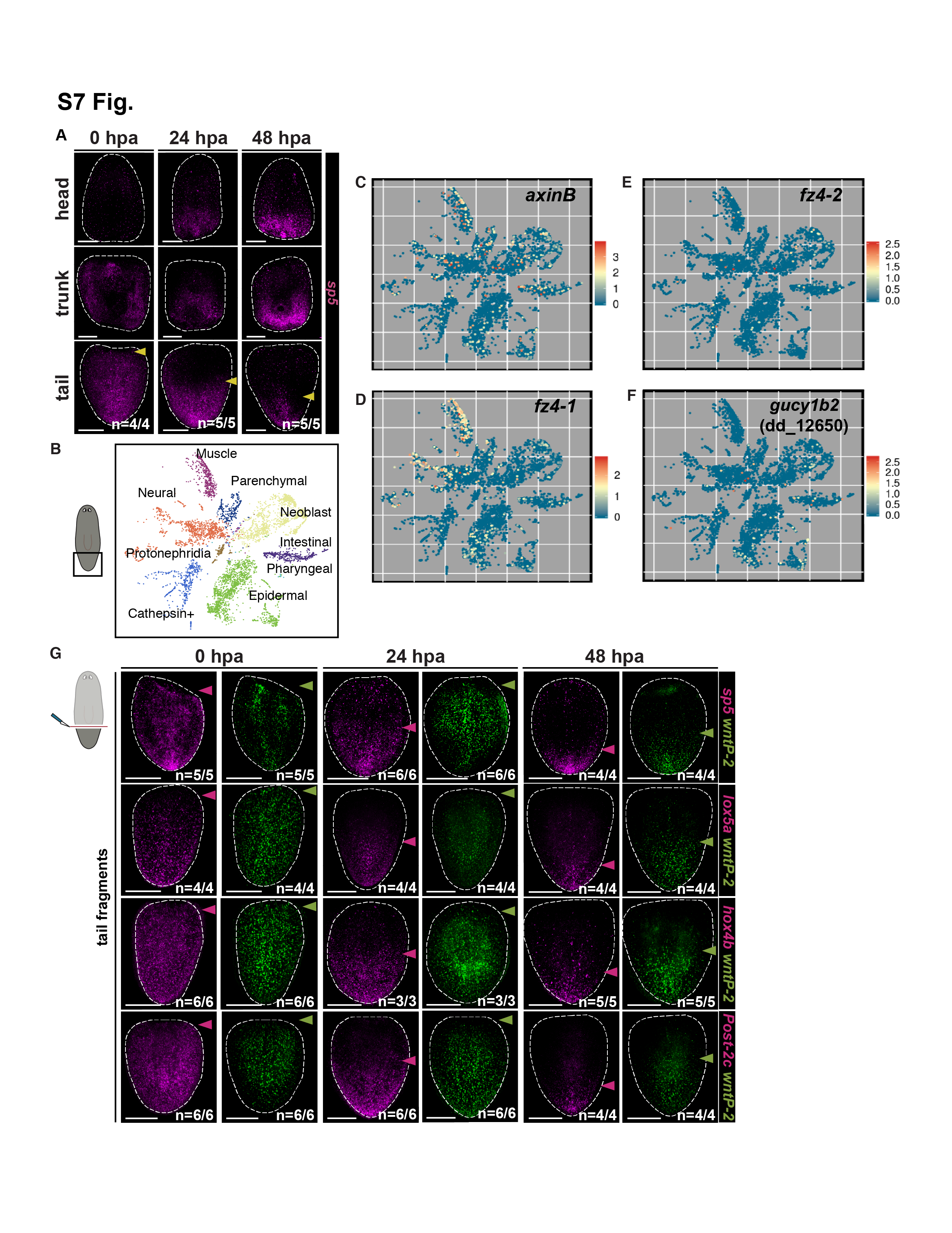

Supplement: S7 Fig — (A) sp5 gene expression changes occur by 24 hours post amputation. FISH for sp5 (magenta) in regenerating heads, trunks, and tails. Yellow arrow indicates anterior boundary of sp5 expression. (B) t-SNE plot key of cells from the planarian tail. Each major tissue type is labelled with a color. (C) tSNE-plot of planarian tail cells colored by axinB gene expression (red, high; blue, low). (D) tSNE-plot of planarian tail cells colored by fz4-1 gene expression (red, high; blue, low). (E) tSNE-plot of planarian tail cells colored by fz4-2 gene expression (red, high; blue, low). (F) tSNE-plot of planarian tail cells colored by gucy1b2 gene expression (red, high; blue, low). (G) Expression of planarian sp5 and Hox genes re-scales prior to wntP-2 re-scaling in regenerating tail fragments. FISH for sp5, lox5a, hox4b, Post-2c (magenta), and wntP-2 (green) at 0, 24, and 48 hours post amputation in regenerating tails. Scale bars, 200μm. (TIF) [file pgen.1008401.s020.tif]

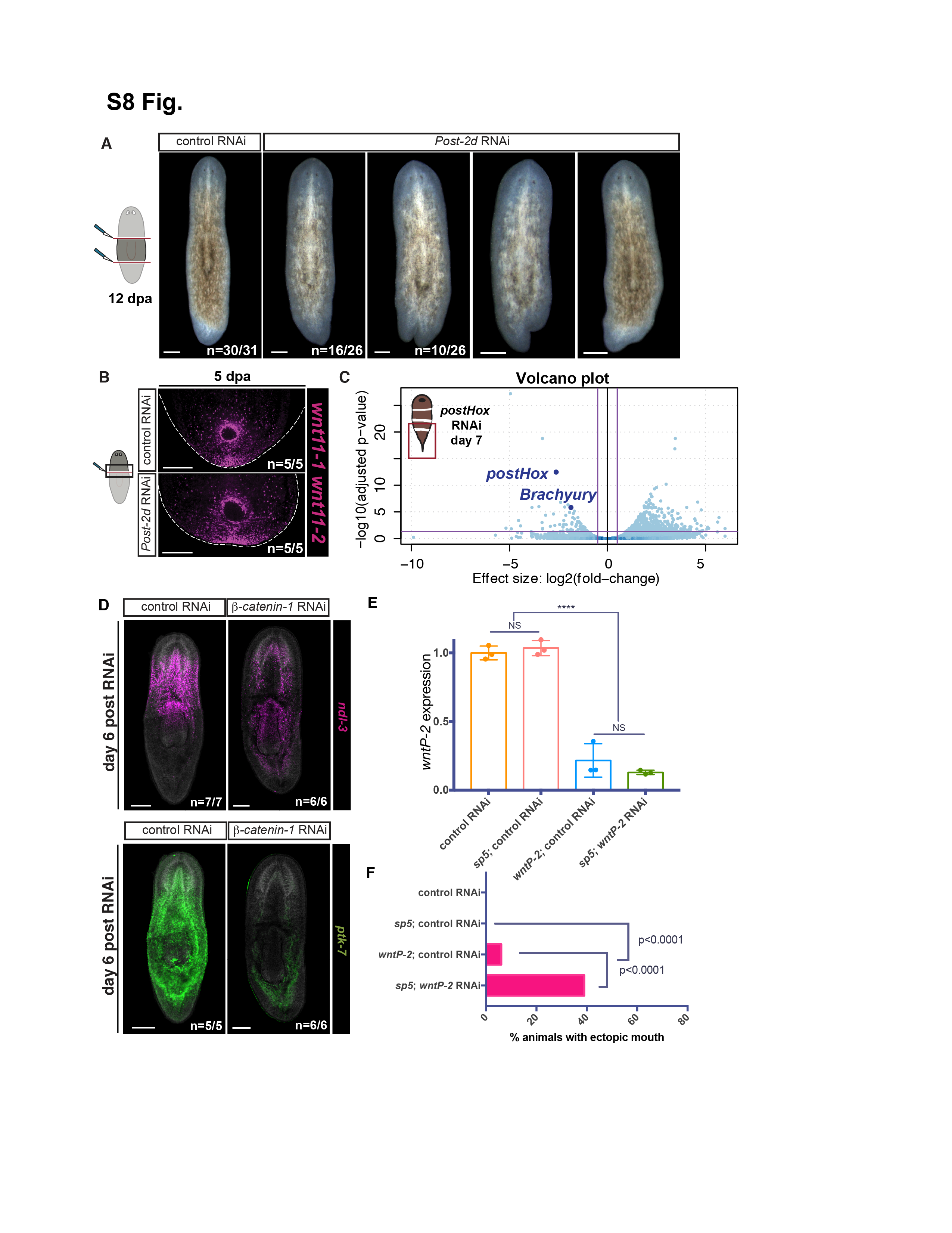

Supplement: S8 Fig — (A) Post-2d RNAi leads to tail-regeneration defects in planarians. Live images of regenerating trunk fragments 12 days after amputation. (B) Post-2d RNAi head fragments express posterior PCGs during regeneration. FISH for wnt11-1 and wnt11-2 pool (magenta) at 5dpa. (C) Hofstenia postHox RNAi leads to down-regulation of Brachyury in the tail. RNA sequencing on Hofstenia tails 7 days after initiation of dsRNA injections for postHox or control RNAi. Volcano plot displays genes differentially expressed between control and postHox RNAi tails with a padj <0.05 and log2 fold change <-1 or >1, Differential expression analysis provided in S7 Table. (D) Expression of ndl-3 and ptk-7 is β-catenin-1 dependent. FISH for ndl-3 (magenta) and ptk-7 (green) 6 days after control and β-catenin-1 RNAi in homeostasis. (E) qRT-PCR for wntP-2 expression in 0 hour trunks after 3 weeks of given RNAi conditions. Data is presented as mean ± S.D. ****p<0.001 (F) Plot shows the percentage of RNAi animals that formed ectopic mouths after 6 RNAi feedings. p-values provided for Fisher’s exact test. 3 independent RNAi experiments are pooled in this analysis. (TIF) [file pgen.1008401.s021.tif]
