# Supplementary material for: A small set of conserved genes, including sp5 and Hox, are activated by Wnt signaling in the posterior of planarians and acoels
Source: PLoS Genet. 2019 Oct 18;15(10):e1008401. doi: 10.1371/journal.pgen.1008401 (PMC6821139; doi:10.1371/journal.pgen.1008401)
Supplement: Attachment — Submitted filename: Tewari_review_responses.pdf [file pgen.1008401.s022.pdf]

Reviewer #1 Wnt signaling plays a vital and highly conserved role in regulating anterior-posterior body patterning across bilaterians. Despite its pivotal role, little is known about the conserved Wnt targets that drive this important developmental process. In this manuscript, Tewari et al., identify the shared Wnt targets responsible for regulating posterior body identity in the planarian Schmidtea mediterranea and the acoel Hofstenia miamia. Specifically, the authors show that Hox genes, the transcription factor sp5, and Wnt signaling components are down-regulated in response to RNAi-mediated b-catenin knockdown in both planarians and acoels. Since acoel worms are Xenacoelomorpha, basal bilaterians separated from planarians by over 550 million years, these newly identified Wnt targets common to both species likely represent a core Wnt-dependent program that is ancestral to all bilaterians. This high-quality work will be of general interest to a broad audience.

Line 307: The authors mention that none of the planarian Hox genes show any observable AP abnormalities after RNAi-mediated knockdown. This group has previously shown that even combinatorial RNAi of three Hox genes, hox4b, post-2c, lox-5a, does not result in AP patterning defect (Scimone et al., 2016). These three genes represent three of the four b-catenin-sensitive Hox genes identified this paper. Does RNAi of all four genes have an effect on AP patterning? Along these lines, does RNAi of the b-catenin target, Hof-Posthox, display any RNAi-mediated AP defects?

To address whether planarian *Hox* genes have a role in AP patterning we attempted individual RNAi of each of the four *Hox* genes identified as *β-catenin*-sensitive in this paper, along with RNAi of all four *Hox* genes together. We found that individual RNAi of *Post-2d* caused tail regeneration defects after amputation. RNAi of all four *Hox* genes together did not lead to any observable phenotype. A new figure (figure 7) has been added with these *Post-2d* RNAi results.

We performed RNA-seq for the *Hof-postHox* gene and found that the expression of another β-catenin target *Brachyury* is down-regulated after *Hof-postHox* RNAi. This data has been added to Figure S8.

Line 312: The images in Figure S5c do not depict Hof-Hox1 and Hof-centHox as being expressed in the "anterior and mid-body" of acoels. Expression seems uniform along the AP axis. Conversely, Hof-Cdx, does not appear to be "expressed broadly on the AP axis of hatchlings" but instead is expressed in a gradient with enrichment in the trunk and tail. The authors should include the expression pattern of the b-catenin-sensitive Hof-postHox.

Hof-Hox1 and *Hof-centHox* are both expressed in the broad anterior-to-mid-body region. Both probes yield weak FISH signal with background signal all over the animal, however the strongest signal is in these regions. We altered the text to say '*Hof-Hox1* and *Hof-centHox* are expressed broadly along the anterior and mid-body of juveniles' to clarify that these are broad expression domains. *Hof-Cdx* is expressed in the trunk and tail, but also has visible expression around the mouth. This sentence has been altered to say 'We also found a *Hofstenia* caudal homolog (*Hof-Cdx*), which was expressed in the trunk and tail of hatchlings, along with some expression in the anterior'. *Hof-postHox* was not detectable by FISH and we have added a sentence about this in the paper.

Line 316: The authors find that planarian and acoel guanylate cyclase homologs “are expressed in the posterior and encode subunits of the GUCY1 family of soluble guanylate cyclase proteins (Fig S6a).” Where is this expression data? Please show in situs in this figure.

Figure call outs have been added for the expression data that was presented in earlier figures (Fig S1F and 4D).

What is the evidence that brachyury does not exist in planarians? Can the authors expand on this point in the manuscript? Are the authors referring to work by María Martín-Durán and Rafael Romero (2011) on S. Polychroa? If so, please cite. Otherwise, authors should show orthology of S. mediterranea Tbx genes (place S. mediterranea Tbx genes on the phylogenetic tree in Fig S6) to support their statement.

This claim was made based on Romero et al., 2011 and independent searches for a *Brachyury* homolog in the *S. mediterranea* genome and transcriptome. The appropriate reference has been added along with the addition of planarian *Tbx* genes to the phylogenetic analysis in Fig S6. This tree shows that all 5 *S.mediterranea Tbx* genes are placed in other Tbx gene families and none in the *Brachyury* family.

Reviewer #2
Wnt signaling is a well-known regulator of the posterior of the body during development and maintenance of many animal species. This manuscript explores whether signaling downstream of Wnt is conserved, using planarians and acoels, organisms that—despite many similarities—are only distantly related. Wnt signaling from muscle cells controls maintenance and regeneration of body polarity in both organisms, and Tewari, et al, use this as a jumping off point for molecular studies. The authors use high throughput sequencing to identify genes differentially regulated after downregulation of beta-catenin in planarians and acoels. They then identify genes that the two lists have in common and zero in on sp5, hox family members, and a small number of other conserved genes. The authors go on to make several other important observations. They show that genes differentially expressed in the tail after beta-catenin perturbation are produced in a variety of cell types (not just muscle). They also show that planarian sp5 has a role in setting the boundary that resides between trunk and tail. Finally, the authors show that acoels possess a brachyury homolog for which expression is altered after beta-catenin(RNAi). This manuscript makes a strong contribution to revealing what happens downstream of Wnt signaling in planarian polarity. Further, it supports the idea that a very well-conserved tail signal (Wnt) affects diverse sets of genes in different animal species, including a small common core of targets. This is a thoughtful and compelling paper that should be of interest to readers of PLoS Genetics. I believe that this paper should be considered for publication, especially if the points below are addressed.

1) I recommend altering the title of the manuscript. The title suggests that the number of genes differentially regulated after perturbation of Wnt signaling in the tail is small. In contrast, the authors show many differentially regulated genes, only some of which are conserved.

The title has been changed to more accurately reflect that the set of conserved Wnt targets is small.

‘A small set of **conserved** genes, including SP5 and Hox, are activated by Wnt signaling in the posterior of planarians and acoels.

2) The authors comparative study would be improved by understanding whether acoel genes identified as being downstream of Wnt (e.g. brachyury) affect tail identity. It especially seems important to know whether sp5 affects polarity in the same or different ways in acoels and planarians.
To address this we performed RNAi for various *Hofstenia* Wnt targets followed by RNA-sequencing. We found that RNAi of *postHox* leads to down-regulation of *Brachyury* expression in the tail by RNA-sequencing. This data has been added to Figure S8. RNAi of *Brachyury* led to a large number of gene expression changes in the posterior, however did not affect any known patterning genes or genes known to be expressed differentially along the AP axis. Additional work on these genes and their targets will be a good subject for future work outside the scope of this manuscript.

We also performed RNAi of *Hof-sp5* followed by RNA sequencing in the tail. We found that *Hof-sp5* RNAi lead to significant up-regulation of two *frizzled* genes in the

tail by RNA sequencing. These results suggest that *Hof-sp5* might inhibit the expression of some Wnt pathway components in the tail. In addition, we found a novel gene (98038232) that is expressed in the anterior and trunk of *Hofstenia* without appreciable expression in the tail. The expression of this gene spread into the tail in *sp5* RNAi animals. This data has been added to Figure 8.

3) The authors emphasize common genes differentially regulated by Wnts in acoels and planarians. One area that was not discussed was the preponderance of genes differentially regulated in the two datasets that were not found to be conserved. I think it would be worth delving into this a bit in the discussion. Is it surprising that so few genes are common? Is it surprising that that the common genes aren't essential for tail identity (at least not as far as the authors have shown)? Do these observations indicate that a conserved Wnt signal from the tail is handled in a species-specific way almost from step one?

The large number of differentially expressed genes that are not conserved might represent the species-specific biology of the posterior in these two evolutionarily distant species. We agree this observation is interesting and a commentary to this effect has been added to the discussion. We performed *Hox* RNAi in planarians and found that the posterior *Hox* gene *post-2d* is important for tail regeneration in planarians. In addition, *sp5* acts with *wntP-2* to restrict trunk identities from the tail. These functional roles suggest these genes are important in the maintenance and/or regeneration of tail identity. *sp5* and posterior *Hox* genes have known roles in tail development in vertebrates. Thus, it is possible a similar Wnt-dependent conserved program is used broadly, and that different additional genes come under the control of this circuit in the course of evolution to establish or maintain diverse features of tails.

4) Accession numbers for datasets and all transcripts/genes should be deposited and also referenced well in the supplemental material. The information seems to be missing from the manuscript in the methods section and many genes (used for RNAi and ISH) are missing from Table S13. Depositing and referencing sequences of interest (for example in Genbank) will prevent mix-ups in the field and will ensure that the authors receive proper acknowledgement for their work.

All gene sequences and RNA-sequencing files (PRJNA558985) have been deposited to NCBI. Accession numbers are provided in supplementary table S13 and methods

Minor points:
1) The timing of the acoel RNAi/RNA-Seq results is later than that of planarians. The authors should describe why they used later time points for this experiment, given that they were focused on identifying the most upstream targets of Wnt signaling. The inclusion criteria for differentially regulated genes also seem to be different and it would be helpful for this decision to be justified in the text.

The rate of tissue turnover during homeostasis has not been thoroughly studied in *Hofstenia miamia.* Therefore it is unclear how quickly one would expect RNAi to affect tissue identity. In addition, it is not known whether *β-catenin-1* RNAi directly

affects tissue identity or requires tissue turnover for a phenotype to occur. Thus we performed *β-catenin-1* RNAi and FISH for known posterior and anterior AP markers at various time points in Figure S4A to determine when we see changes in posterior and anterior identity using this method. We were able to see some changes in AP identity by day 6 post RNAi however these were not robust until a later timepoint (d14). Thus we used an early timepoint (day 3), before we observed any changes by FISH, a time-point at which we began to see some change (day 7) and a late time-point to ensure we captured all robust changes. In the text we have added the sentence 'Wnt-dependent gene expression changes have not been studied in acoels, therefore we first identified early timepoints at which *β-catenin-1* RNAi lead to changes in gene expression in *Hofstenia miamia*.' to clarify why we use PCG expression changes to guide our time point decisions.

A more lenient significance cutoff ($p_{adj}<0.1$ vs. $p_{adj}<0.05$) was used in this species because after *β-catenin-1* RNAi we observed a larger phenotypic variability between animals in acoels compared to planarians by FISH. In addition a log_2FC cutoff was not used in acoels (in planarians log_2FC <-0.5 or >0.5 was applied) because with the chosen p_{adj} cutoff (<0.1) all genes had log_2FC changes that already satisfied this condition (Table S4). A sentence to explain this has been added to the main text – 'A more lenient p_{adj} cutoff was used in *Hofstenia miamia* due to the larger phenotypic variability observed after *β-catenin-1* RNAi compared to planarians.' These data were followed up with an independent method (FISH).

2) The explanations of clusters in the text got somewhat confusing. For example, line 152 "this set" is unclear. Is this from the 52 genes in the cluster or some other subset? Each of the passages describing clusters should be reviewed for clarity.

Passages dealing with descriptions of cluster subsets have been altered for clarity.

3) The sentence in lines 485-6 is unclear. Ubiquitously expressed? Or conserved across all animals?

This sentence was intended to say these Wnt targets may be conserved targets across animals. The sentence 'However, evidence from vertebrates and pre-bilaterians indicates multiple of the Wnt targets in planarians and acoels may be ubiquitous across animals.' has been changed to 'Wnt signaling is involved in primary axis patterning throughout the Metazoa, and some Wnt targets (e.g., *sp5* in Hydra) predate the emergence of bilaterally symmetric phyla. Our findings suggest that a Wnt-dependent program of posterior gene expression including *sp5*, posterior *Hox* genes, *Brachyury*, and Wnt-pathway components was active in the posterior of the last common ancestor of the Bilateria, and will prove to be found broadly in diverse phyla.'

4) The authors should indicate in the figure legends when the FISH images have been inverted.

The sentence ‘Images are presented in grayscale and color inverted’ has been added to the figure legend for Figs 1D; Fig 2D,E; Fig 3C; Fig 4C; S3C,E; S5C.

5) Figures 3b-c might be clearer if channels were shown separately.
We put the individual channels into the supplement.

6) In the top panels of Fig. 5b, it isn’t clear which marker is shown.

Labels have been added to the top panel to indicate which marker is shown.

Reviewer #3

In this work, Tewari et al. combine beta-catenin RNA interference with RNA-sequencing of tail tissues (wherein Wnt activity is high) from planarians and acoels at early time-points post-RNAi treatment. Using this approach, the authors identify a small set of shared genes that are activated by Wnt signaling in the posterior of planarian and acoels, suggesting that a posterior gene expression program is conserved between these organisms. Furthermore, RNAi analysis of sp5 function reveals a role for this gene in the maintenance of proper AP patterning in planarians. This is a solid study and the manuscript is well written. My only major criticism is that the comparative approach taken throughout this study is abandoned for the functional characterization of sp5. The authors should consider performing sp5 RNAi knock down experiments in Hofstenia to test whether there is any obvious phenotype or assess how expression of candidate target genes might be affected as was done for planarians. Analysis of sp5 in acoels or future experiments was omitted from the Discussion.

We performed RNAi of *Hof-sp5* followed by RNA-sequencing in the tail. We found that *Hof-sp5* RNAi lead to significant up-regulation of two *frizzled* genes in the tail by RNA-sequencing. These results suggest that *Hof-sp5* might inhibit the expression of some Wnt pathway components in the tail. In addition, we found a novel gene *spreg-1* that is expressed in the trunk of *Hofstenia* without appreciable expression in the tail. The expression of this gene spread into the tail in *sp5* RNAi animals. This data has been added to Figure 8.

Additional Comments:

1) P. 11: The header and conclusions in this section alternate between stating a Wnt-driven posterior program is conserved in planarians and acoels (as in the title) and making the bolder claim the program is conserved across the Bilateria. I think it is reasonable to propose the Wnt-driven expression programs observed in the paper (especially in the Discussion) may be broadly conserved, but it does not seem accurate to state the mechanism is conserved in [all] Bilaterians from analysis in two organisms. I suggest editing this section and making statement supported by the data, such as the statements in the model (Fig. 6).

The header for this section has been modified to 'A small set of conserved genes are Wnt targets in planarians and acoels'.
The concluding sentence of this section now states: 'These findings suggest that a common and small Wnt-driven posterior program of gene expression including *sp5*, posterior *Hox* genes, *Brachyury*, *guanylate cyclase* genes, and genes encoding Wnt-pathway components themselves, is conserved across distantly related extant bilaterians.'

2) The Discussion is a little unfocused. Although interesting, the paragraphs on Hox and guanylate cyclase genes could be omitted. The discussion on the role of Wnt-driven expression in differentiated tissues and the function of sp5 are limited. I would encourage the authors to discuss the broader implications of their findings. Wnt signaling controls homeostatic self-renewal in several adult tissues in mammals. Is there anything known about the role of sp5 or Hox genes in adult tissue

homeostasis?

The discussion has been re-organized and section headers added to clarify structure. The paragraphs on *Hox* genes and *gucy* genes have been shortened to address this concern. The *Hox* paragraph was retained, because we feel the Wnt regulation of Hox is an important point to highlight and because of the addition of data on the functional role of posterior *Hox's*. We added more discussion on the role of Wnt-driven expression in differentiated tissues as suggested. We did not describe the role of Wnt in regulating stem-cell based renewal, though fascinating, because of our focus specifically on the patterning role for Wnt. There is very little known about sp5 or Hox in adult homeostasis; however we added a sentence about the known regional expression of Hox genes in vertebrate skin.

3) I think the authors have space to include some supplementary data in the main manuscript. Figure S4 could be included in the main paper and perhaps S8 could be appended to Figure 5.
We have now added more data to the main figures and expanded the figures to 9, including the addition of a posterior *Hox* phenotype (now Fig. 7) and *Hof-sp5* RNAi data (Figure 8), and *Hof-postHox* RNAi in Figure S8. We moved some panels from S3 to Figure 2 and two panels of Figure S4 to the main figures.

Minor Edits:

Line 352: "hour" should read "hours"
This has been changed to 'hours'

Line 422-423: "pattern" should read as "patterns" or "a complex pattern"
This has been changed to say 'patterns'

Line 441: "neoblast progenitor"; I think the correct terminology is neoblast progeny. Neoblast progenitor would be something that makes neoblasts.
This sentence has been modified to use the term 'neoblast progeny'.
